# Pristimerin as a Novel Hepatoprotective Agent Against Experimental Autoimmune Hepatitis

**DOI:** 10.3389/fphar.2018.00292

**Published:** 2018-03-28

**Authors:** Dina S. El-Agamy, Ahmed A. Shaaban, Hamdi H. Almaramhy, Sarah Elkablawy, Mohamed A. Elkablawy

**Affiliations:** ^1^Department of Pharmacology and Toxicology, College of Pharmacy, Taibah University, Medina, Saudi Arabia; ^2^Department of Pharmacology and Toxicology, Faculty of Pharmacy, Mansoura University, Mansoura, Egypt; ^3^Faculty of Pharmacy, Aqaba University of Technology, Aqaba, Jordan; ^4^Department of Surgery, College of Medicine, Taibah University, Medina, Saudi Arabia; ^5^Department of Clinical Pharmacy, Faculty of Pharmacy, Tanta University, Tanta, Egypt; ^6^Department of Pathology, College of Medicine, Taibah University, Medina, Saudi Arabia; ^7^Department of Pathology, Faculty of Medicine, Menoufia University, Menoufia, Egypt

**Keywords:** pristimerin, hepatitis, nuclear factor erythroid-related factor 2 (Nrf2), hemeoxygenase-1, nuclear factor kappa-B, apoptosis

## Abstract

Pristimerin (Pris) is bioactive natural quinonoid triterpene that has anti-inflammatory and anti-cancer activities. Meanwhile, its effect against hepatitis needs to be elucidated. This investigation aimed to evaluate the ability of Pris to protect against autoimmune hepatitis (AIH). A mouse model of AIH was established using single concanavalin A (Con A) intravenous injection. Mice were treated with Pris at two different doses (0.4 and 0.8 mg/kg) for 5 days prior to Con A challenge. Markers of hepatic injury, oxidative, inflammatory, and apoptotic damage were estimated. Results have revealed that Pris pretreatment ameliorated Con A-induced hepatic damage. There was decrease in the elevated serum indices of hepatic damage (ALT, AST, ALP, and LDH) and improvement of the histopathological picture of the liver. Pris effectively decreased Con A-induced neutrophil infiltration into the hepatic tissue as presented by amelioration of the level and immuno-expression of myeloperoxidase (MPO). Additionally, Pris attenuated Con A-induced increase in CD4+ T-cells in hepatic tissue. Lipid peroxidation was significantly depressed simultaneously with enhancement of the antioxidant capacity in Pris pretreated animals. Pris also enhanced nuclear factor erythroid 2-related factor 2 (Nrf2) mRNA expression and its binding capacity. In addition, Pris increased mRNA expression of heme-oxygenase-1 (HO-1) and restored its normal level. Furthermore, Pris decreased the level and immuno-expression of nuclear factor kappa-B (NF-κB) as well as the downstream inflammatory cascade (TNF-α, IL-6, and IL-1β). Finally, Pris showed inhibitory effect on Con A-induced apoptotic alteration in liver as it decreased the mRNA expression and levels the apoptotic markers (Bax and caspase-3) and increased mRNA expression and level of the anti-apoptotic protein (Bcl2). In conclusion, this study demonstrates the potent hepatoprotective efficacy of Pris against Con A-induced hepatitis which may be related to anti-oxidative, anti-inflammatory, and anti-apoptotic pathways. Pris could serve as a new candidate for the management of hepatitis.

## Introduction

Autoimmune hepatitis (AIH) is a serious immune mediated inflammatory hepatic disorder that is characterized by massive infiltration of inflammatory cells into the hepatic tissue. This inflammatory condition of liver is associated with elevated serum transaminases and severe hepatic necrosis and apoptosis which may be followed by hepatic cirrhosis and failure (Krawitt, 2006). Concanavalin A (Con A) can induce well known AIH in mice that mimics the pathological picture and underling immune mechanisms of AIH patients (Wang et al., 2012, 2016; Mao et al., 2015). The pathogenesis of Con A-induced acute hepatitis includes several signaling pathways. Con A single injection induces CD4+ T helper cells infiltration into liver tissue contaminant with the release of inflammatory mediators, i.e., tumor necrosis factor alpha (TNF-α) and interleukins (ILs) which contribute to hepatocyte damage (Liu et al., 2012). In addition to CD4+ T cells, neutrophils, natural killer T cells and Kupffer cells essentially contribute to the inflammatory process in Con A-induced hepatitis (Takeda et al., 2000; Bonder et al., 2004). Nuclear factor erythroid-related factor 2 (Nrf2) is another important transcription factor that has been closely linked to inflammation-mediated oxidative damage (Osburn et al., 2006). It has been shown that Con A-induced hepatic injury is associated with inhibiting the Nrf2 signaling pathway which results in down expression of antioxidant enzymes, including HO-1 (Zhao et al., 2015). Recently, many reports elucidated the involvement of nuclear factor kappa-B (NF-κB) in the pathogenesis of Con A-induced AIH. Activation of NF-κB and subsequent up-regulation of downstream inflammatory cascades mediate the inflammatory response in Con A-associated hepatotoxicity (Xu et al., 2008; El-Agamy, 2016; Li et al., 2016). Apoptosis has a crucial role in Con A-induced hepatitis. It was reported that the pro-apoptotic protein, Bax, and Bad are up-regulated in Con A-treated animals whereas the anti-apoptotic protein Bcl2 and Bcl-xl are down regulated leading to hepatocytes apoptosis (Mao et al., 2015).

Pristimerin (Pris) is a major component of different plants of the family Celastraceae. This triterpenoid compound possesses marked anti-oxidative and anti-inflammatory properties (Lu et al., 2010; Larsen et al., 2012; Kim et al., 2013). Pris has antitumor and antiproliferative activities which have been linked to its inhibitory activity on NF-κB activation in tumor cell proliferation (Costa et al., 2008; Lee et al., 2013; Deeb et al., 2014). Furthermore, Pris can suppress proteasome activity, migration of tumor cell, and angiogenesis (Yang et al., 2008; Mu et al., 2012). Additionally, Pris possess potent therapeutic efficacy against autoimmune arthritis in rats. It successfully inhibited tissue damage and inflammation via modulation of inflammatory mediators (Tong et al., 2014). To our knowledge, the ability of Pris to protect the liver against AIH has not been evaluated before. Hence, we tried in the present study to assess the protective effect of Pris against Con A-induced hepatitis and explore the possible pathways underling that effect.

## Materials and methods

### Animals

Male Swiss albino mice (20–22 g) were maintained under standard conditions of humidity, temperature, controlled 12 h dark/light cycle, and free access to standard laboratory rodent food and water. The experimental protocol and procedures were approved by the Research Ethics Committee of Faculty of Pharmacy, Mansoura University, Egypt and the Research Ethics Committee, Taibah University, Saudi Arabia which sticks to “Principles of Laboratory Animals Care” (NIH publication).

### Chemicals and reagents

Pristimerin (Pris) (Tocris Bioscience, Bristol, UK) was dissolved in dimethyl sulfoxide (DMSO, 0.1%). Con A (Sigma-Aldrich, St. Louis, MO, USA) was dissolved in phosphate-buffered saline. Colorimetric kits for alanine aminotransferase (ALT), aspartate aminotransferase (AST), alkaline phosphatase (ALP), and lactate dehydrogenase (LDH) were purchased from Human (Wiesbaden, Germany). Colorimetric kits for malondialdehyde (MDA), superoxide dismutase (SOD), reduced glutathione (GSH), and total antioxidant capacity (TAC) were bought from Spectrum Co. (Cairo, Egypt).

Caspase-3 colorimetric assay kit (Cat. No. BF3100) was obtained from R&D System (USA). Mouse ELISA kits for myeloperoxidase (MPO, Cat. No. EMMPO) and nuclear protein extraction kit (Cat. No. 78833) were purchased from Thermo Fisher Scientific (USA). ELISA kits for TNF-α (Cat. No. MTA00B, R&D System, USA), IL-6 (Cat. No. ELM-IL6-001, RayBiotech, Inc. USA), Bax (Bcl2 Associated X Protein, Cat. No. MBS2509733, MyBioSource Inc., USA), and Bcl2 (Cat. No. E0778m, EIAab, Wuhan, China) were purchased. IL-1β (Cat. No. CSB-E08054m), NF-κB (Cat. No. CSB-E12108m) and heme oxygenase-1 (HO-1, Cat. No. CSB-E08268m) ELISA kits were bought from CUSABIO (Shanghai, China). Nrf2-binding activity was determined using a TransAM Nrf2 kit (Cat. No. 50296, Active Motif, USA).

Anti-CD4 antibody (Cat. No. ab24894) used for flow cytometry and immunohistochemical staining was purchased from Abcam, UK. Primary antibodies against rabbit polyclonal anti NF-κBp65 antibody (Cat. No. RB-9034-P) was purchased from Thermo Fisher Scientific (USA) while that against anti-MPO antibody (Cat. No. ab9535), anti-Bax antibody (Cat. No. ab32503), and anti-Bcl2 antibody (Cat. No. ab59348) was purchased from Abcam, UK. All other chemicals were of standard commercially available biochemical quality.

### AIH model and animal treatment

Based on previous reports, AIH was induced effectively by single intravenous injection of Con A (15 mg/kg) (Chen et al., 2015; Fei et al., 2016). Forty mice were divided into five groups as follows: Control group where mice received intravenous phosphate-buffered saline; Pris group which received Pris (0.8 mg/kg) only; Con A group where mice received an injection of Con A; Pris pretreatment groups (Pris + Con A) where mice were pretreated with two different doses of Pris (0.4, 0.8 mg/kg) for 5 consecutive days prior to Con A injection. Twelve hours after Con A injection, blood, and liver samples were obtained. Serum samples were obtained after blood centrifugation and kept at −80°C for further analysis. Liver samples were flash-frozen and kept at −80°C for PCR analysis. For other set of liver samples, homogenization of liver tissue then centrifugation at for 15 min at 4°C were done to get the supernatants which were kept at −80°C for further analysis.

### Hepatotoxicity indices

Serum ALT, AST, ALP, and LDH were determined under the instruction manual supplied by the manufacturer.

### Histopathology

Piece of liver of each animal was fixed in buffered formalin and embedded in paraffin. Specimen (4–5 μm) were stained with hematoxylin and eosin (H&E). Hepatic lesions in terms of necrosis and inflammation was scored as 0, none; 1, very mild; 2, mild (≤ 30%); 3, moderate (≤ 60%); and 4, severe (≥ 60%) (El-Agamy, 2016).

### MPO measurement

It is used as index for neutrophil infiltration and it was measured in serum according to the instructions of the kit.

### Analysis of CD4+ via flow cytometry

Briefly, the hepatic cell suspension were stained with fluorescence-labeled anti-CD4 antibody for 30 min before washing with phosphate buffered saline (PBS)/1% bovine serum albumin (BSA). The samples were measured using FACS caliber flow cytometer (Becton Dickinson, Sunnyvale, CA, USA) equipped with a compact air cooked low power 15 mwat argon ion laser beam (488 nm). The average number of evaluated nuclei per specimen 20,000 and the number of nuclei scanned was 120 per s. The data obtained was analyzed using computer software (FlowJo, MacVersion 8.84, LLC, Ashland, OR, USA).

### Oxidative stress and antioxidant balance

Levels of MDA, SOD, GSH, and TAC were determined in hepatic homogenates of different experimental groups based on the methods provided by the kits.

### Nrf2-binding activity

Nuclear extracts were used for the determination of Nrf2-binding activity according to the kit's instructions. Briefly, nuclear extract protein (~5 μg) was incubated in a 96-well plate containing the immobilized consensus Nrf2-binding site. After washing the wells for three times, detection of bound Nrf2 was done spectrophotometrically at 450 nm using Nrf2 antibody and secondary antibody conjugated with horseradish peroxide.

### Real-time PCR analysis

Initially, total RNA was extracted from liver samples using RNeasy Mini kit (Qiagen, USA). The RNA quality was checked using the ratio A260/A280 and pure RNA samples were converted to cDNA using Quantitect Reverse Transcription Kit as per manufacturer's instructions (Qiagen, USA). PCR was done using SYBR Green based on the instruction manual (Qiagen, USA). The primers sequences were as follows: Nrf2 (Mouse) 5′-AAGAATAAAGTCGCCGCCCA-3′ (forward); 5′-AGATACAAGGTGCTGAGCCG-3′ (reverse), HO-1 (Mouse) 5′-GAAATCATCCCTTGCACGCC-3′ (forward); 5′-CCTGAGAGGTCACCCAGGTA-3′ (reverse), Bax (Mouse) 5′-GATCCAAGACCAGGGTGGCT-3′ (forward); 5′-TCCCCCATTCATCCCAGGAA-3′ (reverse), Caspase-3 (Mouse) 5′-GAGCTTGGAACGGTACGCTA-3′ (forward); 5′-GAGTCCACTGACTTGCTCCC-3′ (reverse), Bcl2 (Mouse) 5′-GCGTCAACAGGGAGATGTCA-3′ (forward); 5′-CCCAGAATCCACTCACACCC-3′ (reverse), β-actin (Mouse) 5′-AGCTGAGAGGGAAATCGTGC-3′ (forward); 5′-CTTCTCCAGGGAGGAAGAGGA-3′ (reverse). The relative mRNA expression of different genes was quantified using the ΔΔCt method. β-actin was used as housekeeping gene.

### Immunohistochemistry (IHC)

IHC staining was performed automatically using Ventana Bench Mark XT system (Ventana Medical Systems, Tucson, AZ). The liver sections were immuno-stained using primary antibodies: rabbit polyclonal antibody to MPO, CD4+ T-cells, NF-κBp65, Bax, and Bcl2 following the same procedures described previously (El-Agamy, 2016).

### ELISA assay

Levels of HO-1, NF-κB, TNF-α, IL-6, IL-1β, Bax, caspase-3, and Bcl2 were determined in the liver following the instructions of the kits.

### Statistical analysis

The results are expressed as mean values and standard error (SE). One way ANOVA followed by a *post hoc* Tukey Kramer Multiple comparison test were used to compare different groups. The scale of histopathological examination was analyzed using non-parametric Kruskal–Wallis test followed by Dunn's test. Statistical significance was defined at a value of *p* < 0.05.

## Results

There was no significant difference between control and Pris groups in all of the measured parameters.

### Effect on hepatotoxicity indices and histopathological examination of the liver

**Figure 1:**
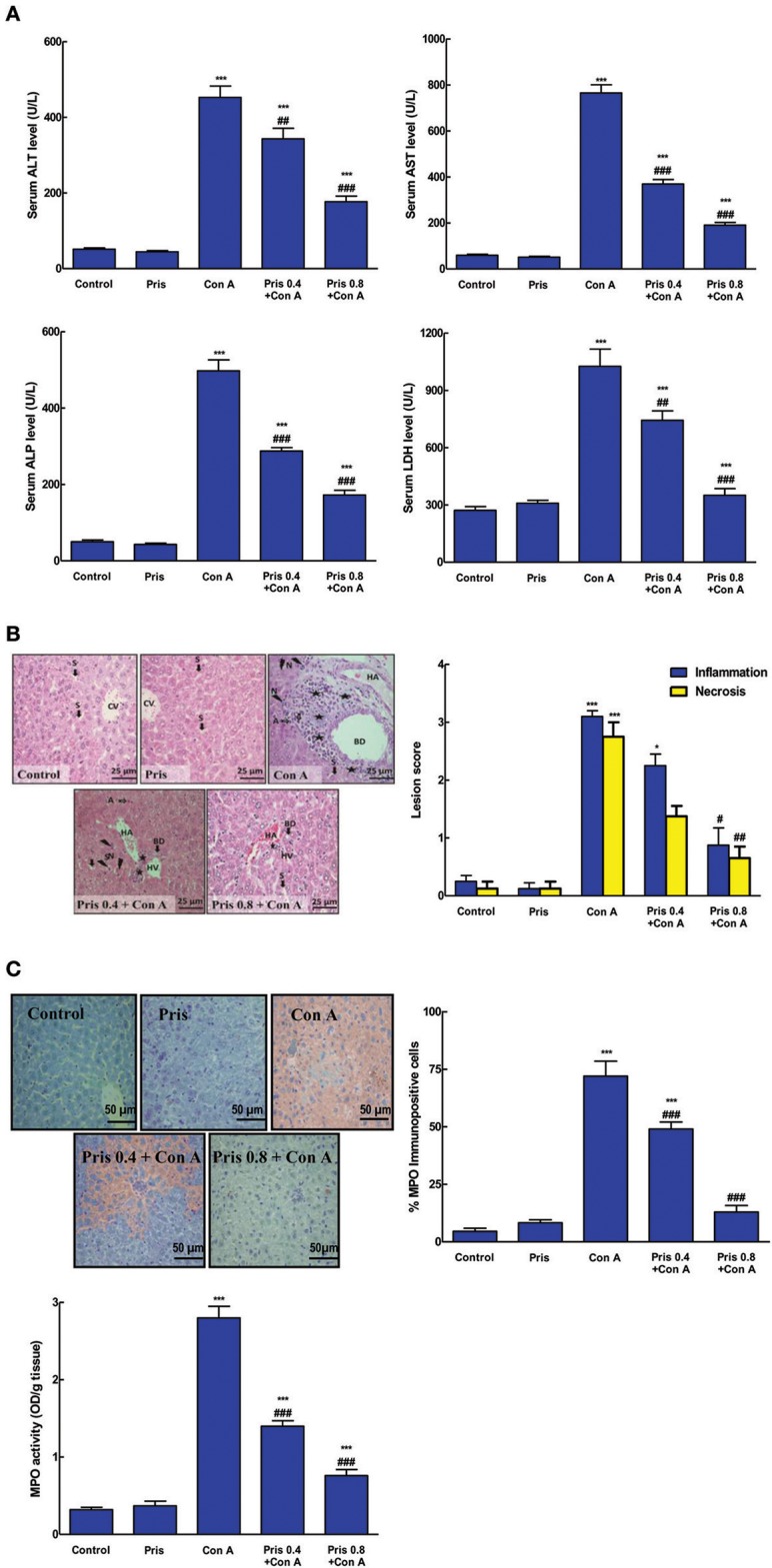
Effects of pristimerin (Pris) on Con A-induced hepatotoxicity. **(A)** Serum alanineaminotransferase (ALT), aspartate aminotransferase (AST), alkaline phosphatase (ALP), lactate dehydrogenase (LDH), and gamma-glutamyl transferase (GGT). **(B)** Pathological specimen of liver stained with H&E (×400): Control and Pris groups showed normal hepatic architecture (normal CV central vein and S blood sinusoids). Con A injection induced marked pathological damage in the portal area in the form of cloudy swelling, necrosis (N: lightning bolt) and apoptosis (A: notched arrows) and periportal (BD, bile duct; HA, hepatic artery; and HV, hepatic veins) infiltration with inflammatory cells mainly lymphocytes (Stars), while Pris pretreated animals showed improvement of these lesions. Histopathological inflammation and necrosis scores in hepatic tissue of different experimental groups. **(C)** Hepatic myeloperoxidase (MPO) immunoexpression (200×); Hepatic MPO activity. Data are the mean ± SEM (*n* = 8). *P < 0.05, ***P < 0.001 vs. control group; ^#^*P* < 0.05, ^##^*P* < 0.01, ^###^*P* < 0.001 vs. Con A group.

As shown in Figure 1A, Con A injection elevated serum markers of hepatotoxicity as ALT, AST, ALP, and LDH compared to control animals. This elevation was significantly ameliorated in Pris pretreated animals compared to Con A group. That effect was most prominent in the group receiving the higher dose of Pris contaminant with Con A.

Histopathological results were in the same line with the biochemical ones. Liver specimen of the control animals showed normal hepatic tissue with no signs of any lesions. Con A intoxicated mice exhibited liver damage in the form of massive neutrophil infiltration, congestion, necrosis, inflammation, cloudy swelling, and apoptosis. The necrosis and inflammation scores were significantly increased compared to the control specimen (Figure 1B). However, Pris pretreatment succeeded to suppress the development of Con A-induced lesions and greatly improved all of the above mentioned pathological signs.

### Effect on MPO

MPO level is a known indirect indicator for neutrophils recruitment in case of inflammation. Con A administration significantly increased the level and immuno-expression of MPO compared to normal animals (Figure 1C). Pris pretreatment dramatically attenuated the MPO level and immuno-expression compared to Con A group.

### Effect on CD4+ T cells infiltration into the liver tissue

**Figure 2:**
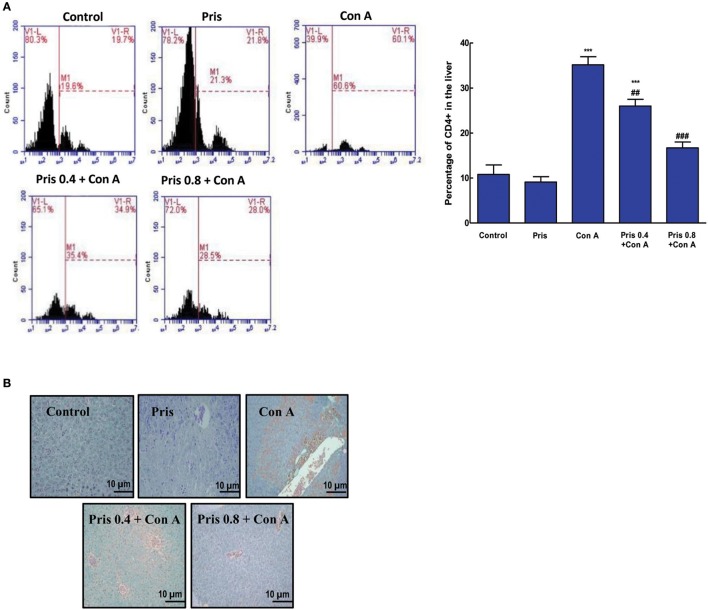
Influence of Pris on Con A-induced recruitment and activation of CD4+ into the hepatic tissue **(A)**. Representative flow-cytometric histograms of different groups **(B)**. IHC staining of CD4+ in the hepatic tissue (200x) showing negative immuno-staining for CD4+ cells in the control while high immuno-staining for CD4+ cells in Con A group. Pris pretreated groups exhibited much lower immuno-staining for CD4+. Data are the mean ± SEM (*n* = 8). ***P < 0.001 vs. control group; ^##^*P* < 0.01, ^###^*P* < 0.001 vs. Con A group.

Based on flow cytometry and IHC staining results, Con A injection induced a significant increase in the infiltrating CD4+ T cells into the hepatic tissue compared to the control animals. The percentage of CD4+ T cells was significantly decreased in livers of Pris pretreated animals and even returned to normal levels compared to Con A group (Figures 2A,B).

### Effect on oxidative stress and antioxidant parameters

**Figure 3:**
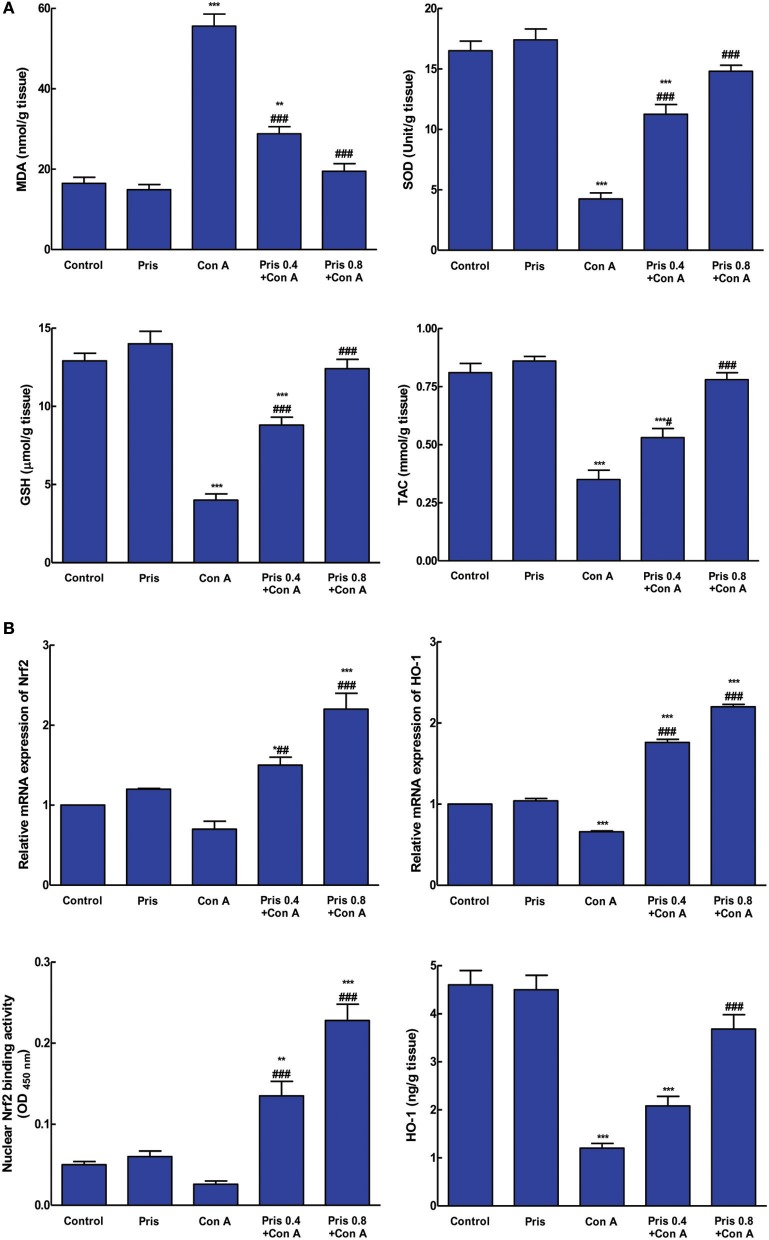
Effects of pristimerin (Pris) on Con A-induced alterations in **(A)**. Oxidative stress and antioxidant indices: MDA, Malondialdehyde; SOD, Superoxide dismutase; GSH, Reduced glutathione; TAC, Total antioxidant capacity; **(B)**. Nuclear factor erythroid-related factor 2 (Nrf2) mRNA expression and binding activity; heme-oxygenase-1 (HO-1) mRNA expression and level in hepatic tissue. Data are the mean ± SEM (*n* = 8). *P < 0.05, **P < 0.01, ***P < 0.001 vs. control group; ^##^*P* < 0.01, ^###^*P* < 0.001 vs. Con A group.

Con A injection induced elevation of MDA content contaminant with depression of SOD, GSH, and TAC in the hepatic tissue. Pris pretreatment alleviated these alteration and restored the normal levels of SOD, GSH, and TAC compared to Con A group (Figure 3A).

### Effect on Nrf2 and HO-1 level

As presented in Figure 3B, Nrf2 mRNA expression and binding activity weren't significantly depressed in Con A group compared to control one. However, Pris pretreatment significantly enhanced Nrf2 mRNA expression and binding activity compared to Con A group. Additionally, Con A significantly decreased the mRNA expression and level of HO-1 compared to control group. Pris pretreatment enhanced mRNA expression of HO-1 and increased HO-1 level compared to Con A group.

### Effect on NF-κB and inflammatory cytokines

**Figure 4:**
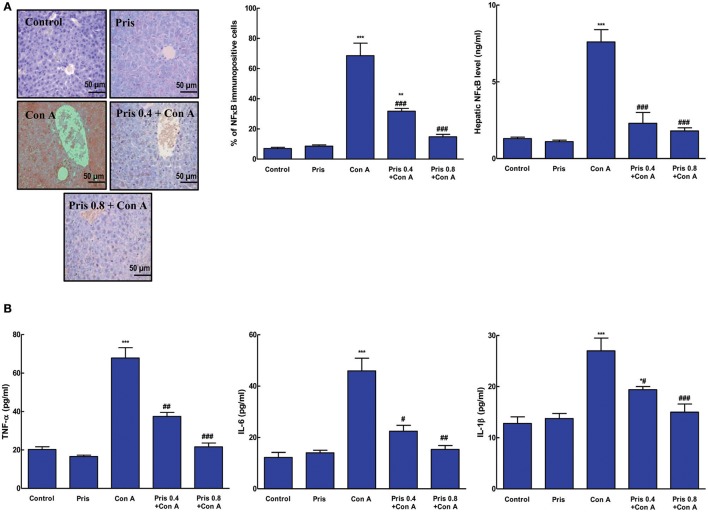
Effect of pristimerin (Pris) on Con A-induced activation of nuclear factor kappa-B (NF-κB) and cytokine release in liver. **(A)** Immunohistochemical staining of nuclear NF-κB p65 (200×): Control and Pris groups showed minimal light brown nuclear immuno-staining while Con A group exhibited high level of brown nuclear immuno-staining of NF-κB. Pris treated animals showed reduction in the intensity of the immunoexpression of NF-κB. Semiquantitative analysis of NF-κB immunohistochemical staining results in liver tissues of different groups, expressed as % of NF-κB immunopositive cells. Level of NF-κB in the hepatic tissue. **(B)** Cytokine levels (TNF-α, IL-6, IL-1β). Data are the mean ± SEM (*n* = 8). *P < 0.05, **P < 0.01, ***P < 0.001 vs. control group*;* ^#^*P* < 0.05, ^##^*P* < 0.01, ^###^*P* < 0.001 vs. Con A group.

As presented in Figure 4A, Con A administration resulted in elevation of the level of NF-κB and increased immuno-expression of NF-κBp65 in the hepatic tissue compared to normal animals. Pris pretreatment alleviated the level of NF-κB and suppressed its activation compared to Con A group. Additionally, animals of Con A group expressed high levels of inflammatory cytokines (TNF-α, IL-6, IL-1β) in the hepatic tissue compared to the control group. Pris + Con A groups showed great attenuation of the levels of these inflammatory cytokines compared to Con A group (Figure 4B).

### Effect on apoptosis markers

**Figure 5:**
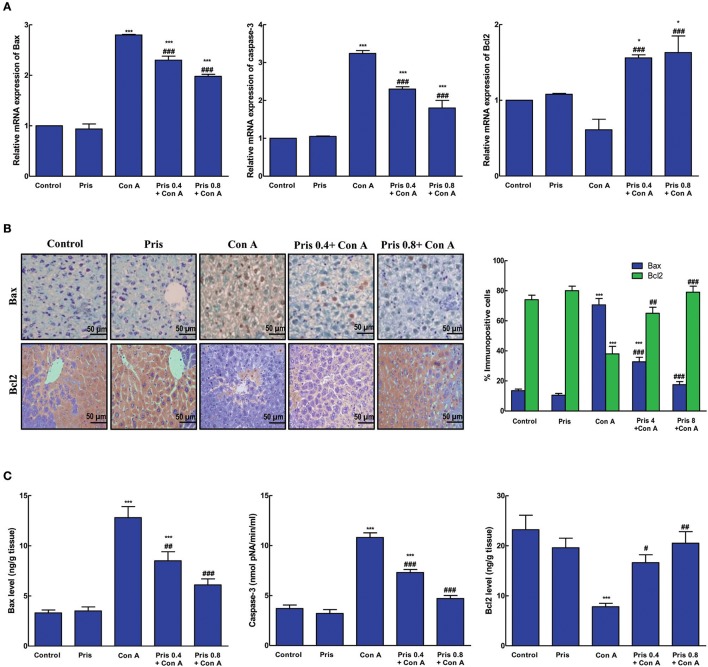
Effects of pristimerin (Pris) on Con A-induced apoptosis. **(A)** Relative mRNA expression of Bax, caspase-3 and Bcl2. **(B)** Immuno-expression of Bax and Bcl2 in the hepatic tissue (200×) showing negative nuclear immuno-staining of Bax and high cytoplasmic expression of Bcl2 in both control and Pris groups while Con A group expressed high brown nuclear immuno-staining of Bax and low nuclear brown staining of Bcl2. Pris pretreatment attenuated Con-induced expression of Bax as represent by reduced nuclear brown staining and enhanced the cytoplasmic immunoexpression of Bcl2**. (C)** Levels of Bax, caspase-3 and Bcl2 in the hepatic tissue. Data are the mean ± SEM (*n* = 8). *P < 0.05, ***P < 0.001 vs. control group; ^#^*P* < 0.05, ^##^*P* < 0.01, ^###^*P* < 0.001 vs. Con A group.

Con A induced elevation in mRNA and immuno-expression of Bax as well as its level. Similarly, mRNA expression and level of caspase-3 significantly increased in the hepatic tissue of Con A group. In addition, Con A significantly decreased the immuno-expression and level of the anti-apoptotic protein, Bcl2, compared to control group. Pris pretreatment counteracted Con A-induced changes in the expression and levels of these apoptotic indices. Pris significantly attenuated the mRNA expression and hepatic levels of Bax and capase-3 simultaneously with decrease in Bax immuno-expression. Pris also caused significant elevation in mRNA and immuno-expression of Bcl2 as well as its hepatic level (Figure 5).

## Discussion

Pristimerin (Pris) is a natural triterpenoid derivative that was shown primarily to inhibit tumor angiogenesis (Costa et al., 2008; Yang et al., 2008). Recently, Pris has been reported to possess potent protective properties against multiple inflammatory disorders such as experimental arthritis, LPS-induced inflammatory response and allergic airway inflammation (Kim et al., 2013; Hui et al., 2014; Tong et al., 2014; Deng et al., 2015; Jin et al., 2016). Based upon these previous positive data, the current study was designed to test whether Pris has hepatoprotective activity against experimental AIH or not? Also, we explored how this activity is mediated through. The results of the study have revealed for the first time the potent protective activity of Pris against Con A-induced hepatitis which may be mediated through anti-inflammatory, antioxidant, and anti-apoptotic pathways.

Con A, lectin obtained from *Canavalia brasiliensis*, is used to establish the best experimental model for immunological hepatitis that closely resemble that of the human (Wang et al., 2012). Con A-induced AIH is rapid and severe, it is sufficient to investigate the inflammatory response associated with severe liver damage in mice 8–12 h after a single dose of Con A (Heymann et al., 2015; Wang et al., 2016). The current results revealed that Con A injection induced marked hepatic injury as manifested by high serum levels of ALT, AST, ALP, and LDH. This was further confirmed by deteriorated histopathological lesions of the liver. Additionally, Con A injection resulted in neutrophils infiltration as indicated by increased level and immuno-expression of MPO in the hepatic tissue. The deleterious effects of Con A on liver have been similarly shown in previous investigations (Feng et al., 2008; Mohamed et al., 2014; El-Agamy, 2016). Noteworthy, Pris pretreatment reversed all of these Con A-induced biochemical and histopathological alterations leading to marked improvement of the hepatic function which indicates potent hepatoprotective activity of Pris.

In the next step, we tried to clarify the possible molecular pathways involved in the hepatoprotective effect of Pris. CD4+ T cells have been shown to play a fundamental role in conducting liver inflammation in Con A-induced hepatitis. Mice lacking CD4+ T cells were fully protected against Con A-induced hepatitis (Tiegs et al., 1992). Following Con A injection, CD4+ T-cells recruit into the liver, become activated and induce inflammatory reaction and inflammatory mediators release in the bloodstream (Carambia and Herkel, 2010; Darwish et al., 2015; Xue et al., 2015). In this context, agents that can suppress CD4+ T-cells infiltration could possess protective activity against Con A-induce hepatitis. Results of this study indicated the high level of hepatic CD4+ T-cells following Con A injection. On the other hand, Pris pretreatment reduced the number of infiltrating CD4+ T indicating that CD4+ T-cell can be considered as therapeutic target of Pris.

Oxidative stress is considered as cornerstone of various liver injury. Reactive oxygen species (ROS) overproduction and associated lipid peroxidation are known to increase the expression of inflammatory cytokines leading to acute inflammation and injury. Furthermore, ROS modulate intracellular signaling pathways and the expression of nuclear transcription factors as NF-κB. Elevation of ROS and suppression of antioxidant system have been reported to be involved in mediating Con A-induced hepatitis probably secondary to immune inflammatory response in liver (Shirin et al., 2010; El-Agamy, 2016; Wang et al., 2016). The current results confirmed the accumulation of oxidative stress marker, MDA, contaminant with depressed GSH, SOD, and TAC in case of Con A administration. However, Pris enhanced the antioxidant system and restored the normal levels of GSH, SOD, and TAC with resultant repressed lipid peroxidation of the hepatocytes. The potent inhibitory effect of Pris on ROS generation has been demonstrated before in a model of LPS-induced inflammatory response (Hui et al., 2014).

Nuclear factor erythroid 2-related factor 2 (Nrf2) is a transcription factor that regulates the transcription of a battery of cytoprotective genes in response to oxidative/electrophilic stress. Under normal conditions, Nrf2 is present in inactive form in the cytoplasm as it is sequestered by kelch-like ECH associating protein 1 (Keap1). In oxidative stressful condition, Nrf2 is released from Keap1 and translocates into the nucleus where it induces the expression of cytoprotective genes such as HO-1 (Klaassen and Reisman, 2010). HO-1 is a stress protein involved in maintaining cellular homeostasis and it can be stimulated by various factors including oxidative stress and inflammatory conditions. HO-1 overexpression can confer cyto-protection against oxidative stress induced cellular damage (Min et al., 2011). Recent reports have focused on the protective role of HO-1 against Con A-induced hepatitis and how HO-1 is being considered as one of the important targets for antioxidant therapeutics (Gambhir et al., 2014; Zhao et al., 2015). Our results have shown that although Con A did not significantly affect Nrf2 expression and binding capacity in hepatic tissue although it significantly decreased the level and expression of HO-1. Earlier clinical studies have shown that HO-1 expression is greatly depressed in HCV-infected patients which was attributed to the ability of HCV core gene products to modulate HO-1 expression (Abdalla et al., 2004). However, the depressed level of HO-1 in our AIH model group may provide new evidence that the etiology and pathogenic signaling of AIH may involve suppression of HO-1 expression. Noteworthy, this point needs further molecular elucidation. Results additionally clarified that Pris treatment markedly enhanced Nrf2 expression and binding capacity as well as the expression and level of HO-1 indicating that Pris antioxidant activity may be mediated through Nrf2/HO-1 potentiation. This could be in part responsible for the hepatoprotective activity of Pris.

The NF-κB pathway is a key mediator of the cellular response to various extracellular stimuli and its inhibition may result in protection against tissue injury. In normal state, the transcription factor, NF-κB, is present in the cytoplasm in an inactive form. Con A has been shown to activate NF-κB which regulates the inflammatory process as it induces the expression and the release of inflammatory cytokines such as TNF-α and ILs (Feng et al., 2008, 2009; Xue et al., 2015; El-Agamy, 2016). Previous reports have focused on the great impact of TNF-α on Con A-induced hepatitis and how it is linked to activation of other cytokines (Mizuhara et al., 1994; Shirin et al., 1998; Sharma et al., 2013). Con A induces overproduction of TNF-α which amplify the inflammatory response in hepatitis. Additionally, TNF-α induces the release other cytokines leading to up-regulation of adhesion molecules and enhancement of cell activation (Bonizzi and Karin, 2004; Wang et al., 2016). Also, TNF-α through its binding to its receptors can induce hepatocyte apoptosis or necrosis (Shirin et al., 2010). In addition, IL-6 has been found to have a role in acute liver inflammation following Con A administration (Tagawa et al., 2000). Inhibition of IL-6 signaling has been reported to prevent Con A-induced hepatitis (Malchow et al., 2011; Sharma et al., 2013; Darwish et al., 2015). The results of present study confirmed the activation of NF-κB and subsequent release of inflammatory cytokines (TNF-α, IL-6, IL-1β) upon Con A administration. However, Pris pretreatment markedly hindered NF-κB activation and lowered its level to the control value. Also, Pris pretreatment alleviated the release of these inflammatory markers indicating potent anti-inflammatory effect of Pris against Con A-induced hepatitis. These data are in line with the previous ones which showed the inhibitory action of Pris against the release of inflammatory cytokines either *in vivo* or *in-vitro* (Kim et al., 2013; Tong et al., 2014; Deng et al., 2015; He et al., 2016).

Previous studies have shown that Con A injection induces hepatic apoptosis which is linked to the high expression of pro-apoptotic protein caspases-8 and Bax (Li et al., 2011). Recently, depression of the anti-apoptotic proteins, as Bcl2 and Bcl-xl, and elevation of pro-apoptotic proteins, as Bax and Bad, have been demonstrated in Con A treated animals (Mao et al., 2015). Alterations of pro-apoptotic and anti-apoptotic proteins in Con A treated animals have been linked to variability in Nrf2 and NF-κB signaling pathways which leads to hepatocyte apoptosis (Gonzalez-Rodriguez et al., 2014). Our results confirmed previous findings as Con A increased apoptosis in the liver. That was evident through the enhanced expression and levels of Bax and caspase-3 simultaneous with depressed immuno-expression and level of Bcl2. Interestingly, Pris reversed Con A-induced alterations in these apoptotic markers. Pris ameliorated the expression and level of Bax and caspase-3 and increased the expression and level of Bcl2. These data afford rational to presume that the ability of Pris to modulate the Nrf2/NF-κB pathways could suppress hepatocyte apoptosis and leads to improvement of hepatic injury.

Collectively, our data demonstrate the potent anti-inflammatory and antioxidant activities of Pris which may be linked to activation of Nrf2/HO-1 pathway and inhibition of NF-κB mediated inflammatory pathway leading to attenuation of Con A-induced liver damage and apoptosis. So, Pris can be considered as a novel hepatoprotective candidate against AIH. Additional studies are needed for further elucidation of the hepatoprotective effects of Pris.

## Author contributions

All authors are equally contributed to all aspects of the study. ME performed histopathological and immunohistopathological analysis.

### Conflict of interest statement

The authors declare that the research was conducted in the absence of any commercial or financial relationships that could be construed as a potential conflict of interest.
